# Aβ43 levels determine the onset of pathological amyloid deposition

**DOI:** 10.1016/j.jbc.2023.104868

**Published:** 2023-05-29

**Authors:** Marc D. Tambini, Tao Yin, Metin Yesiltepe, Lionel Breuillaud, Simone P. Zehntner, Cristina d'Abramo, Luca Giliberto, Luciano D'Adamio

**Affiliations:** 1 Department of Pharmacology, Physiology & Neuroscience, New Jersey Medical School, Brain Health Institute, Jacqueline Krieger Klein Center in Alzheimer's Disease and Neurodegeneration Research, Rutgers, The State University of New Jersey, Newark, New Jersey, USA; 2 Biospective Inc, Montreal, Quebec, Canada; 3 Litwin-Zucker Center for the Study of Alzheimer’s Disease and Memory Disorders, Feinstein Institutes for Medical Research, Institute of Molecular Medicine, Northwell Health System, Manhasset, New York, USA; 4 Institute of Neurology and Neurosurgery, Northwell Health System, Manhasset, New York, USA

**Keywords:** γ-secretase, Alzheimer’s disease, amyloid-β, amyloid plaques, amyloid precursor protein, APP, Knock–In rat, Presenilin

## Abstract

About 2% of Alzheimer’s disease (AD) cases have early onset (FAD) and are caused by mutations in either *Presenilin*s *(PSEN1/2)* or *amyloid-β precursor protein (APP*). PSEN1/2 catalyze production of Aβ peptides of different length from APP. Aβ peptides are the major components of amyloid plaques, a pathological lesion that characterizes AD. Analysis of mechanisms by which PSEN1/2 and APP mutations affect Aβ peptide compositions lead to the implication of the absolute or relative increase in Aβ42 in amyloid-β plaques formation. Here, to elucidate the formation of pathogenic Aβ cocktails leading to amyloid pathology, we utilized FAD rat knock-in models carrying the Swedish *APP* (*App*^*s*^ allele) and the *PSEN1 L435F* (*Psen1*^*LF*^ allele) mutations. To accommodate the differences in the pathogenicity of rodent and human Aβ, these rat models are genetically engineered to express human Aβ species as both the Swedish mutant allele and the WT rat allele (called *App*^*h*^) have been humanized in the Aβ-coding region. Analysis of the eight possible FAD mutant permutations indicates that the CNS levels of Aβ43, rather than absolute or relative increases in Aβ42, determine the onset of pathological amyloid deposition in FAD knock-in rats. Notably, Aβ43 was found in amyloid plaques in late onset AD and mild cognitive impairment cases, suggesting that the mechanisms initiating amyloid pathology in FAD knock-in rat reflect disease mechanisms driving amyloid pathology in late onset AD. This study helps clarifying the molecular determinants initiating amyloid pathology and supports therapeutic interventions targeting Aβ43 in AD.

Mutations in the *amyloid-β precursor protein* (*APP*) and *Presenilin 1/2* (*PSEN1/2*) genes cause Familial forms of Alzheimer’s disease (FAD). APP processing can result in the formation of amyloid β (Aβ). PSEN1/2 are the catalytic components of the γ-secretase complex, a protease that cleaves Aβ from APP-βCTF, a polypeptide generated by β-secretase processing of APP. Aβ peptides of different lengths are generated by two γ-secretase–dependent product lines consisting of four sequential COOH→NH2-terminal trimming steps. In the first catalytic step, APP-βCTF is processed by γ-secretase at two ε-sites generating two membranes bound Aβ fragments (Aβ49, product line 1 and Aβ48, product line 2). Aβ49 is converted into Aβ46 (second catalytic step), which is converted into Aβ43 (third catalytic step). Finally, Aβ43 is trimmed into Aβ40 (fourth catalytic step). In product line 2, catalytic steps 2 to 4 originate the following peptides from Aβ48: → Aβ45 → Aβ42 → Aβ38 (1). The efficiency of these catalytic events depends on the activity and processivity of γ-secretase, and reduced processivity can lead to increased production of longer Aβ peptides at the cost of shorter Aβ species.

The widely accepted amyloid cascade hypothesis postulates that accumulation of Aβ42, which has higher hydrophobicity and is more prone to aggregation than Aβ40, in oligomeric forms and amyloid plaques is the main pathogenic trigger of Alzheimer's disease (AD) (2). The genetic evidence from FAD cases, that is, that FAD mutations are found in the genes coding for the Aβ precursor substrate and an Aβ generating protease, is consistent with a pathogenic role of Aβ. The evidence that *APP* and *PSEN1/2* pathogenic mutations alter Aβ production further supports the amyloid cascade hypothesis. For instance, a double pathogenic mutation in APP, occurring in a Swedish family, just NH_2_-terminal to the β-secretase cleavage site, favors β-processing of APP and production of APP-βCTF and causes a strong increase of Aβ generation in humans (3) as well as knock-in mouse (4) and rat (5) models. In contrast, a genetic variant just carboxyl-terminal to the β-site that reduces β-processing of APP and, consequently, reduces Aβ generation in humans (6) and in a knock-in rat model (7) protects against AD (6). *PSEN1* and *PSEN2* mutations reduce the activity and processivity of γ-secretase (8), leading to an alteration of the ratios between short and long forms of Aβ, in favor of long Aβ forms (9, 10).

In this pathogenic framework, understanding the essential changes in the molecular composition of Aβ profiles that initiate amyloid pathology in AD could point to the Aβ species that should be therapeutically targeted to delay, prevent, or revert amyloid pathology. Most studies have analyzed changes in Aβ42 and/or Aβ40 production, pointing to either absolute or relative (to Aβ40) increments in Aβ42 levels as a pathogenic hallmark in AD (10, 11, 12, 13, 14, 15, 16, 17, 18, 19). Still, few studies have come to opposite conclusions (20, 21). These contradictions may be the result of a narrow focus on Aβ40 and Aβ42, since FAD mutations can significantly alter the generation of other Aβ species, such as Aβ38 and Aβ43 (8, 22, 23, 24, 25).

Model organisms expressing FAD mutations in a physiological manner could help dissect mixtures of Aβ species that favor amyloid pathology. To test this hypothesis, we have utilized two rat knock-in models of FAD. One model carries the aforementioned Swedish *APP* mutation (*App*^*s*^ rat) (5); the other carries the *PSEN1 L435F* mutation (*Psen1*^*LF*^ rat) (26). To accommodate the possibility of differences in pathogenicity of rodent and human Aβ, these rat models are genetically engineered to express human Aβ species as both the Swedish mutant allele and the WT rat allele (*App*^*h*^) have been humanized in the Aβ-coding region. *App*^*s*^ knock-in rats recapitulate the biochemical changes of human APP-Swedish metabolism (3, 27, 28) and produce significantly higher levels of human Aβ38, Aβ40, and Aβ42 (5). Consistent with *in vitro* and *in vivo* evidence (16, 23, 24, 29), the *PSEN1 L435F* mutation causes a significant loss of γ-secretase activity and processivity in rats. *Psen1*^*LF*^ rats reproduced previously reported alterations in Aβ species (24, 30), which include a reduction in total amyloid production with minimal levels of Aβ38 and Aβ40, while concentrations of Aβ43 are significantly increased (26).

In this study, we tested the compound effects of these mutations with the purpose of gaining insights into the composition of pathogenic Aβ cocktails leading to amyloid pathology. We utilized eight rat knock-in FAD models carrying the following permutations of the pathogenic Swedish *APP* and *PSEN1 L435F* mutations: (1) *App* Swedish heterozygous; (2) *App* Swedish homozygous; (3) *Psen1 L435F* mutant heterozygous; (4) *Psen1 L435F* mutant homozygous; (5) *App* Swedish and *Psen1 L435F* mutant double heterozygous; (6) *Psen1 L435F* mutant homozygous and *App* Swedish heterozygous; (7) *Psen1 L435F* mutant heterozygous and *App* Swedish homozygous; (8) *App* Swedish and *Psen1 L435F* mutant double homozygous. As explained above, the control group used in our experiments also produces human Aβ species, thereby allowing to differentiate the effects of APP and PSEN1 pathogenic mutation from the effects of human Aβ *versus* rodent Aβ.

Although rodent AD models have been extensively used to address this question, the nine rat models we used offer the following advantages over previous models that should increase the translatability of the results to human AD. (1) Contrary to classical transgenic models, these rat models are genetically faithful to the human disease. Mutant genes and humanized *App* are expressed under the control of the natural endogenous promoter and gene locus. Therefore, APP/PSEN1 expression and APP processing occur following a physiological, quantitative, spatial-temporal, and cell-specific pattern. (2) These knock-in models carry *App* and *Psen1* mutations naturally occurring in human FAD cases. In contrast, many transgenic models, and the *App*^*NL-F*^ and *App*^*NL-G-F*^ knock-in rodents (4, 31), express chimeric alleles carrying two or more pathogenic mutations in *APP* that in humans occur independently. (3) Many transgenic models, and the *App*^*NL-G-F*^ knock-in rodents, carry mutations in the Aβ region that increase the propensity to form protofibrils compared with WT human Aβ. Thus, these models cannot properly inform about pathogenic Aβ cocktails leading to amyloid pathology in late onset AD (LOAD) patients. (4) Transgenic AD models co-express human Aβ (either WT or mutant) together with rodent Aβ. Since human Aβ shows increased propensity to form pathogenic oligomers and protofibrils compared with rodent Aβ and considering that rodent Aβ can alter oligomerization kinetics of human Aβ by forming mixed oligomers, the copresence of rodent and human Aβ is a confounding factor when studying pathogenic Aβ cocktails leading to amyloid pathology in LOAD patients. In contrast, the rat knock-in models used here express only WT human Aβ. In summary, the unprecedented number of models analyzed in this study and the sole expression of WT human Aβ at physiological levels allows to correlate nine distinct patterns of human Aβ species cocktails to pathogenic amyloid deposition.

Finally, to determine whether these FAD knock-in rat models reflect disease mechanisms driving amyloid pathology in sporadic AD, we analyzed amyloid plaques composition in LOAD and mild cognitive impairment (MCI) cases.

## Results

### Knock-in rats harboring *App*^*s*^ mutant alleles plus one *Psen1*^*LF*^ allele do not develop amyloid pathology at 18 months of age

**Figure 1:**
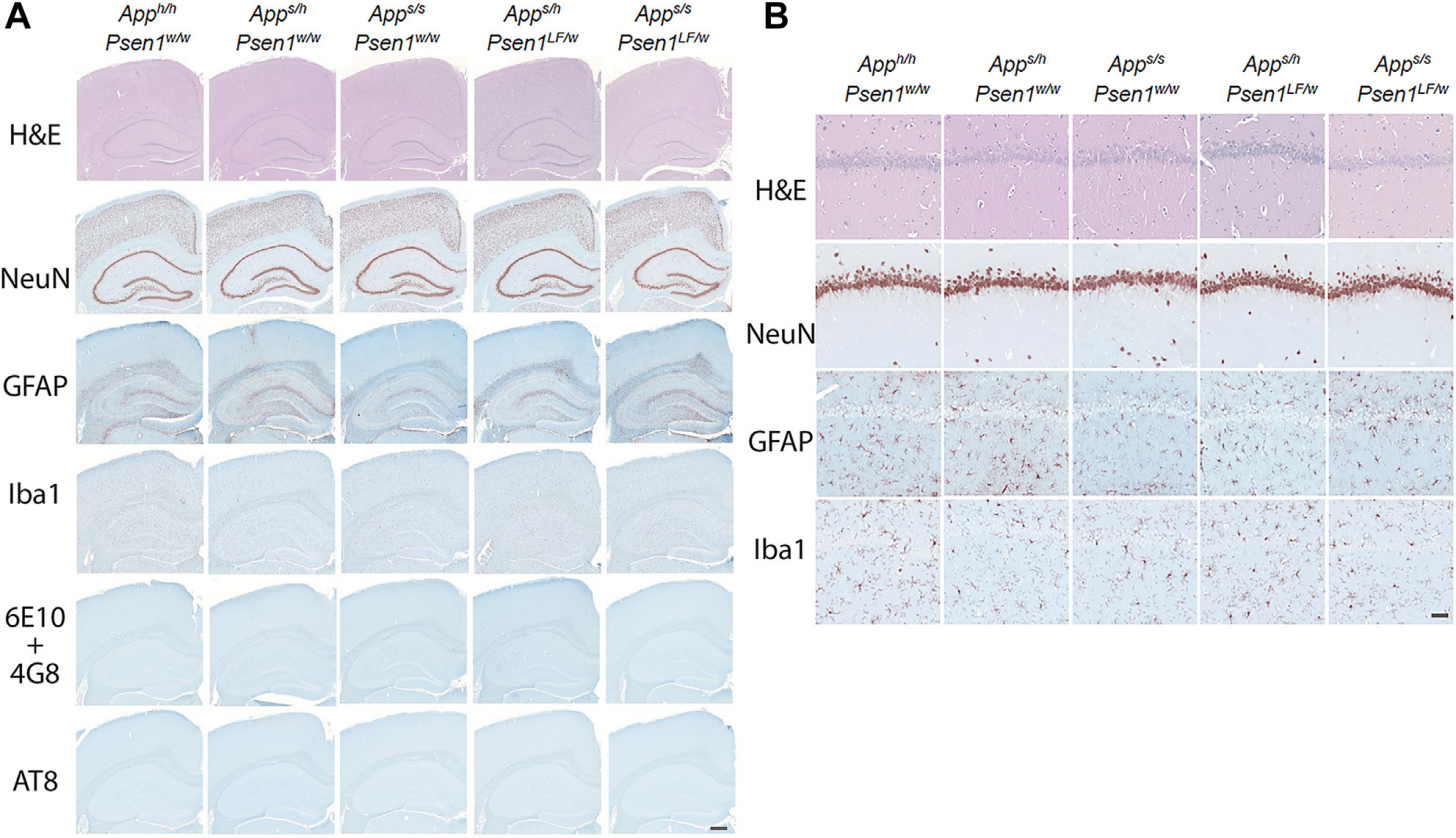
**Histopathological analysis of 18-month-old knock-in rats with different combinations of *App* and *Psen1* mutant alleles.***A*, representative images of the anterior hippocampus and overlaying somatosensory cortex of rat brains. Illustrates of, from the *top* to *bottom*, NeuN, GFAP, Iba1, amyloid-beta (6E10+4G8), and phospho-Tau (AT8) staining, respectively. The scale bar is equivalent to 500 microns. *B*, high-magnification picture of the hippocampal CA1 subregion for the samples depicted in (*A*). Illustrates of, from the *top* to *bottom*, NeuN, GFAP, Iba1, amyloid-beta (6E10+4G8), and phospho-Tau (AT8) staining, respectively. The scale bar is equivalent to 50 microns. APP, amyloid precursor protein; PSEN, Presenilin.

*App*^*s*^ and *Psen1*^*LF*^ knock-in rats recapitulate the changes in APP processing and human Aβ production caused by these pathogenic mutations in patients. *App*^*s*^ rats generate significantly higher levels of APP-βCTF and, consequently, its γ-cleavage products Aβ peptides (5). In contrast, *Psen1*^*LF*^ knock-in rats generate high levels of Aβ43 but minimal levels of Aβ38 and Aβ40, with a reduction in total amyloid production (26), confirming that this FAD mutation causes a significant loss of γ-secretase activity and processivity. For both knock-in lines, changes in Aβ species are gene-dosage–dependent. To determine whether interactions of these two pathogenic mutations prompt amyloid-β pathology, we crossed *App*^*s/h*^*:Psen1*^*LF/w*^ to *App*^*s/h*^*:Psen1*^*w/w*^ rats to generate *App*^*h/h*^*:Psen1*^*w/w*^ (our control group, 2 males and 2 females), heterozygous and homozygous Swedish (*App*^*s/h*^*:Psen1*^*w/w*^ and *App*^*s/s*^*:Psen1*^*w/w*^, 2 males and 2 females for each genotype), double heterozygous (*App*^*s/h*^*:Psen1*^*LF/w*^, 2 males and 2 females), Swedish homozygous and *Psen1*^*LF*^ heterozygous (*App*^*s/s*^*:Psen1*^*LF/w*^, 3 males and 1 female) rats. Brains were harvested from ∼18-month-old rats and analyzed by immunohistochemistry (IHC). H&E staining was utilized to assess the tissue quality and overall brain structure. NeuN staining was used to assess neuronal density in the cortical mantle and neuronal cell layers in the hippocampus. GFAP staining was used to assess the activation level of the astrocytes and the presence of neuroinflammation. Iba1 staining was used to assess the activation state of microglia and the potential presence of inflammatory foci. The 6E10 and 4G8 antibodies, which target amino acids 1 to 17 and 18 to 23 of Aβ, respectively, were mixed and used to evaluate amyloid plaques. Tau phosphorylation was evaluated with the AT8 antibody. Representative images of dorsal hippocampus and sensory cortex and of magnified area of the CA1 are shown in Figure 1, *A* and *B*, respectively.

H&E staining illustrates very similar structures within these brain regions for all knock-in animals (Fig. 1, *A* and *B*). Like the observations made with the H&E-stained sections, no overt neuronal loss was observed with NeuN staining (Fig. 1, *A* and *B*). In GFAP staining, no dense clusters of astrocytes were apparent in 18-month-old knock-in rat, and an even distribution of GFAP expression was observed throughout the brain (Fig. 1, *A* and *B*). Overall, there is no evidence of a widespread astrocytic inflammatory response related to the genotype. Iba1 staining showed no evidence of a clear microglial inflammatory response related to the genotype, that is, a feature consistent in most of the samples of the group (Fig. 1, *A* and *B*). Throughout the brain, the morphology of the microglia is generally ramified, without patches of bushy activated microglia or inflammatory foci, and both female and male cohorts have very similar Iba1 staining patterns. The 6E10 + 4G8 antibodies did not show any sign of amyloid pathology in these 18-month-old knock-in rats (Fig. 1, *A* and *B*). Finally, AT8 antibody showed no staining in any of the animals studied (Fig. 1, *A* and *B*).

### Gene-dosage–dependent amyloid pathology in *App*^*s*^*: Psen1*^*LF*^ mutant knock-in rats

*Psen1 L435F* homozygote mutant mice are perinatally lethal (16) in a manner that resembles the early embryonic lethality of *Psen1* KO mice (32). This is likely the result of the PS1 L435F–mediated disruption of Notch signaling. Unexpectedly, we found that homozygote *App*^*h/h*^*:Psen1*^*LF/LF*^ rats are born in Mendelian ratios, survive into adulthood, and have preserved neurodevelopment and Notch signaling, despite altered APP metabolism (26). Based on this evidence, we reexamined whether interactions of these two pathogenic mutations cause amyloid pathology, focusing our attention on genotypes carrying two mutant *Psen1*^*LF*^ alleles (*App*^*h/h*^*:Psen1*^*LF/LF*^, *App*^*s/h*^*:Psen1*^*LF/LF*^, and *App*^*s/s*^*:Psen1*^*LF/LF*^) in addition to *App*^*h/h*^*:Psen1*^*w/w*^ control animals and *App*^*h/h*^*:Psen1*^*LF/w*^ rats that were not tested at 18 months of age. Given that homozygous *Psen1*^*LF*^ mutant rats show increased postnatal lethality (26), animals were tested at 14 months of age. We analyzed the following rat subjects: *App*^*h/h*^*:Psen1*^*w/w*^, 7 males and 7 females; *App*^*h/h*^*:Psen1*^*LF/LF*^, 2 males and 4 females; *App*^*s/h*^*:Psen1*^*LF/LF*^, 6 males and 6 females; *App*^*s/s*^*:Psen1*^*LF/LF*^, 8 males and 2 females; *App*^*h/h*^*:Psen1*^*LF/w*^, 2 males and 3 females.

**Figure 2:**
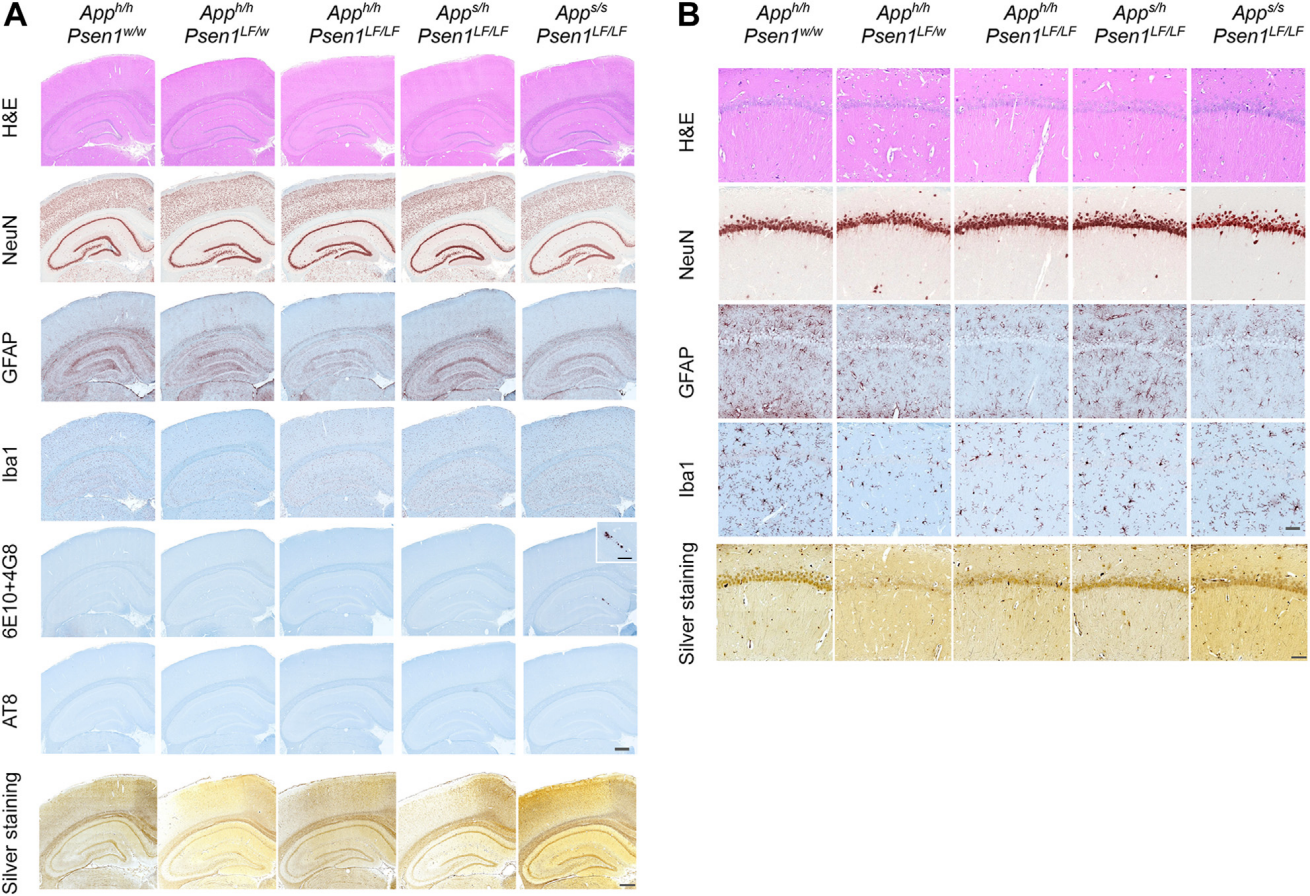
**H&E, NeuN, GFAP, Iba1, 6E10 + 4G8 (amyloid β), AT8 (pTau), and modified Bielschowsky’s silver staining of 14-month-old knock-in rats carrying *App***^***s***^**and *Psen1***^***LF***^**FAD mutations.** Staining was performed in rats of the following genotypes (number of female and male rats used are indicated in parentheses): *App*^*h/h*^*:Psen1*^*w/w*^ (7 females and 7 males), *App*^*h/h*^*:Psen1*^*LF/w*^ (5 females and 1 male), *App*^*h/h*^*:Psen1*^*LF/LF*^ (2 females and 4 males), *App*^*s/h*^*:Psen1*^*LF/LF*^ (6 females and 6 males), and *App*^*s/s*^*:Psen1*^*LF/LF*^ (8 female and 2 males) knock-in rats. *A*, representative images in *App*^*h/h*^*:Psen1*^*w/w*^, *App*^*h/h*^*:Psen1*^*LF/w*^, *App*^*h/h*^*:Psen1*^*LF/LF*^, *App*^*s/h*^*:Psen1*^*LF/LF*^, and *App*^*s/s*^*:Psen1*^*LF/LF*^ rats are illustrated from *left* to *right*. The scale bar is equivalent to 500 microns. An inset in the 6E10 + 4G8 *App*^*s/s*^*:Psen1*^*LF/LF*^ panel shows higher magnification of small hippocampus plaques (the scale bar is equivalent to 200 microns). *B*, detailed images of the H&E, NeuN, GFAP, Iba1, and modified Bielschowsky’s silver staining in the dorsal hippocampus-CA1. The scale bar is equivalent to 50 microns. APP, amyloid precursor protein; FAD, Familial forms of Alzheimer’s disease; PSEN, Presenilin.

Representative images of H&E staining of dorsal hippocampus and sensory cortex (Fig. 2*A*) and of magnified area of the CA1 illustrate very similar structures within these brain regions for all knock-in rats (Fig. 2*B*). A comparison of the male and female cohorts did not show evidence of a gender-driven difference. One *App*^*h/h*^*:Psen1*^*LF/LF*^ sample, subject 437 (S437), presented hydrocephalus, and *App*^*h/h*^*:Psen1*^*LF/w*^ S439 had a large tumor compressing the hypothalamus. Otherwise, all the subjects showed no evidence of gross structural changes in comparison to the control *App*^*h/h*^*:Psen1*^*w/w*^ subjects. No apparent neurodegeneration-related defects were evident in these animals and there was no evidence of pyknotic stressed or dying neurons.

**Figure 3:**
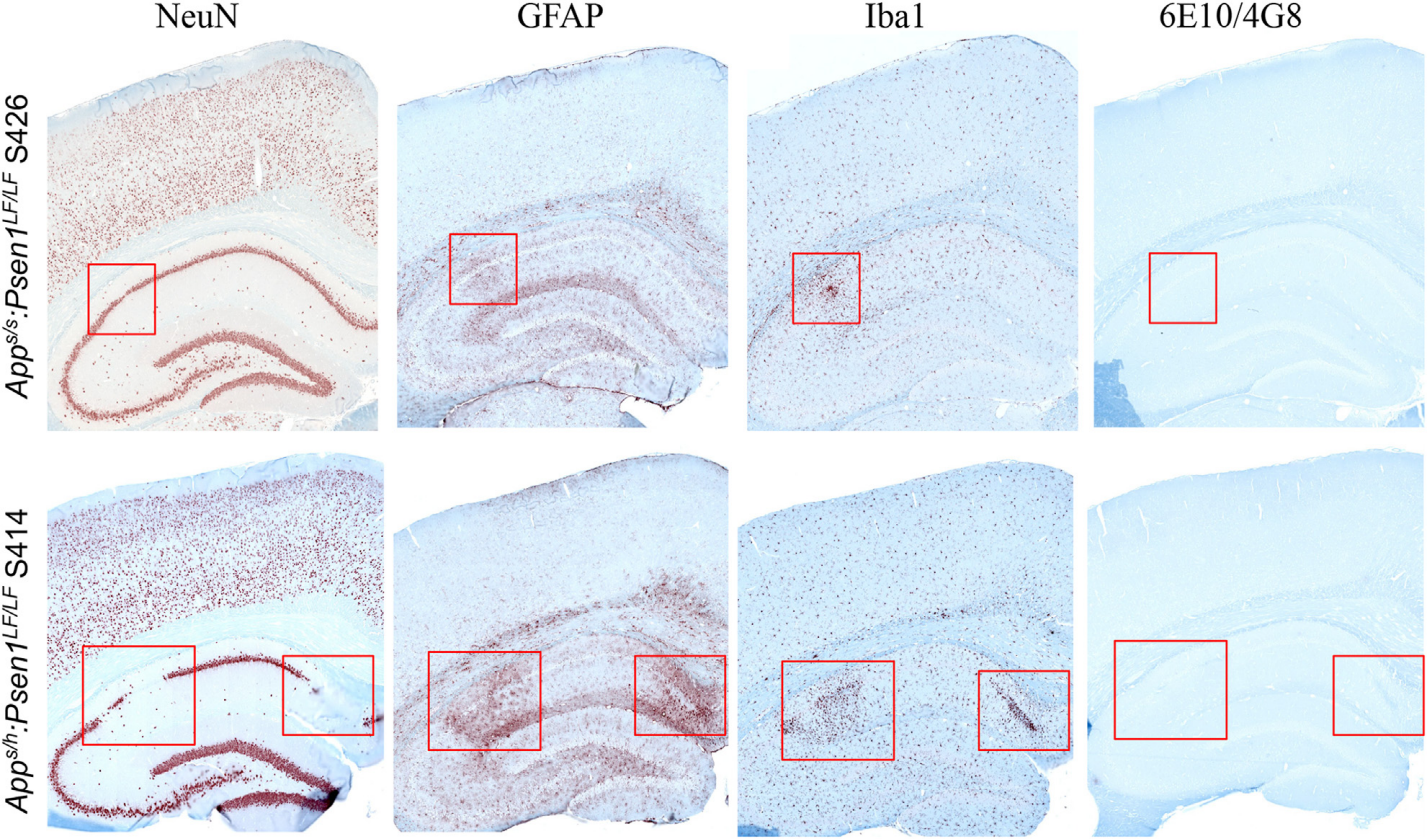
**Representative pictures illustrating hippocampal neurodegeneration in two samples, *App***^***s/s***^***:Psen1***^***LF/LF***^**S426 and *App***^***s/h***^***:Psen1***^***LF/LF***^**S414**. NeuN, GFAP, IBA1, and amyloid staining performed on consecutive sections in *App*^*s/s*^*:Psen1*^*LF/LF*^ S426 and *App*^*s/h*^*:Psen1*^*LF/LF*^ S414. Area of neurodegeneration is shown with a *square*. The scale bar is equivalent to 500 microns. APP, amyloid precursor protein; PSEN, Presenilin.

No overt neuronal loss was observed in the NeuN-stained sections. A representative subject from each of the groups studied is illustrated in Figure 2*A*. Magnified area of the CA1 also illustrates very similar structures within the hippocampus for all knock-in groups (Fig. 2*B*). Blinded qualitative analysis confirmed there was no obvious difference in NeuN density among the nine groups (see Table 1). However, *App*^*s/h*^*:Psen1*^*LF/LF*^ S414 and *App*^*s/s*^*:Psen1*^*LF/LF*^ S426 presented focal neurodegeneration with pyknotic cells, in the CA1 layer of the dorsal hippocampus (Fig. 3).

**Table 1:** Qualitative scoring of IHC

Groups	NeuN	Iba1	GFAP
*App*^*h/h*^*:Psen1*^*w/w*^	2.06 ± 0.2	2.25 ± 0.25	2.35 ± 0.41
*App*^*h/h*^*:Psen1*^*LF/w*^	1.91 ± 0.18	2.12 ± 0.28	2.5 ± 0.63
*App*^*h/h*^*:Psen1*^*LF/LF*^	1.70 ± 0.22	2.2 ± 0.3	1.83 ± 0.37
*App*^*s/h*^*:Psen1*^*LF/LF*^	1.91 ± 0.18	2.02 ± 0.07	2.12 ± 0.74
*App*^*s/s*^*:Psen1*^*LF/LF*^	2.05 ± 0.5	2.35 ± 0.34	2.06 ± 0.46

Summary of the qualitative assessment performed on the NeuN, Iba1, and GFAP IHC. Assessments were performed on a 0 to 3 scale (1 = low, 2 = average, 3 = high), with 0.5 increments, on the dorsal hippocampus and the cortex above; the mean and the SD are shown. No qualitative evaluation was performed in the P- Tau, AT8 samples due to the absence of staining nor in the H&E and silver stains due to the lack of perceivable discriminative features.

No dense clusters of astrocytes were apparent with GFAP staining in the majority of 14-month-old knock-in rat, and an even distribution of GFAP expression was observed throughout the brain. A representative subject from each genotype is illustrated in Figure 2*A*. A magnified area of the CA1 also illustrates very similar astrocytic activation within the hippocampus for the FAD mutant knock-in rat groups compared to the control animals (Fig. 2*B*). Overall, there is no evidence of a widespread astrocytic inflammatory response related to the genotype. The only exceptions were clear astrocytic activation surrounding the CA1 focal degeneration in *App*^*s/h*^*:Psen1*^*LF/LF*^ S414 and *App*^*s/s*^*:Psen1*^*LF/LF*^ S426 (Fig. 3), three animals with widespread activation, *App*^*w/w*^*:Psen1*^*LF/w*^ S438/S439 and *App*^*s/s*^*:Psen1*^*LF/LF*^ S418 (suspected to be related to the presence of a pituitary tumor, not shown). Microglial foci and areas found with activated microglia (see below) did not appear to be associated with astrocytic activation. Both the female and male cohorts have very similar GFAP staining patterns. Blinded qualitative analysis confirmed there was no obvious difference in astrocytic activation within the knock-in and WT groups (Table 2).

**Table 2:** Summary of the pathological features observed in all the groups

Groups	Focal hippocampal neurodegeneration	Iba1 foci	Iba1 activation region	Amyloid plaques
*App*^*h/h*^*:Psen1*^*w/w*^	0/14 (0%)	0/14 (0%)	0/14 (0%)	0/14 (0%)
*App*^*h/h*^*:Psen1*^*LF/w*^	0/6 (0%)	1/6 (16.6%)	1/6 (16.6%)	0/6 (0%)
*App*^*h/h*^*:Psen1*^*LF/LF*^	0/6 (0%)	1/6 (16.6%)	1/6 (16.6%)	0/6 (0%)
*App*^*s/h*^*:Psen1*^*LF/LF*^	1/12 (8.3%)	3/12 (25%)	2/12 (16.6%)	2/12 (16.6%)
*App*^*s/s*^*:Psen1*^*LF/LF*^	1/10 (10%)	3/10 (30%)	2/10 (20%)	10/10 (100%)

Summary of the pathological features observed in all the groups studied, the frequency of occurrence is expressed as percentage.

**Figure 4:**
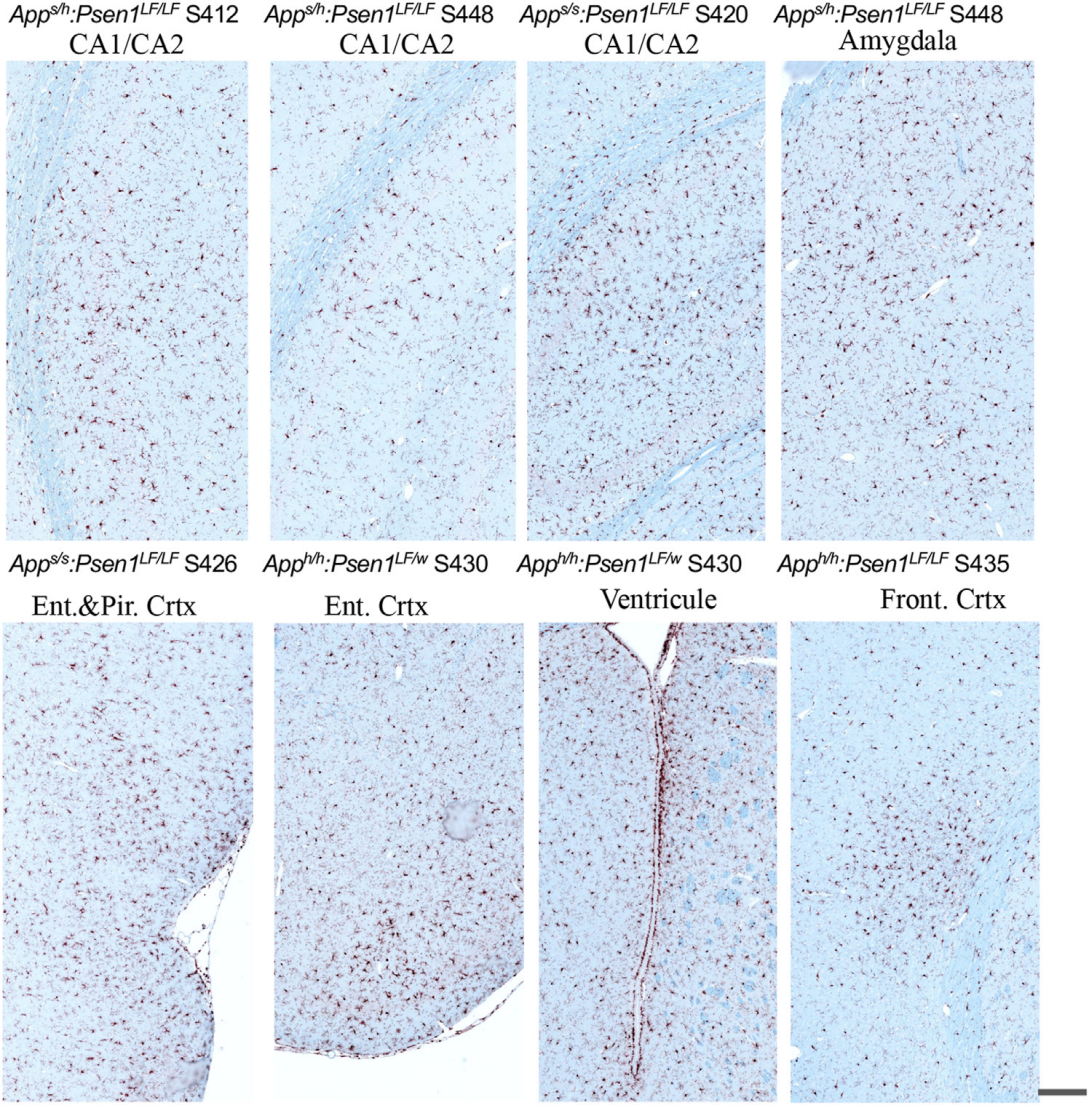
**Representative areas with IBA1 microglial activation magnification in knock-in rats *App***^***s/s***^***:Psen1***^***LF/LF***^**S420 and S426, *App***^***s/h***^***:Psen1***^***LF/LF***^**S412, S448, *App***^***h/h***^***:Psen1***^***LF/w***^**S430 and *App***^***h/h***^***:Psen1***^***LF/LF***^**S435.** Representative images of the IBA1 feature called “microglial activation” (*red-brown chromogen*) in knock-in rats. All pictures were taken at the same magnification; the scale bar is equivalent to 200 microns. APP, amyloid precursor protein; PSEN, Presenilin.

**Figure 5:**
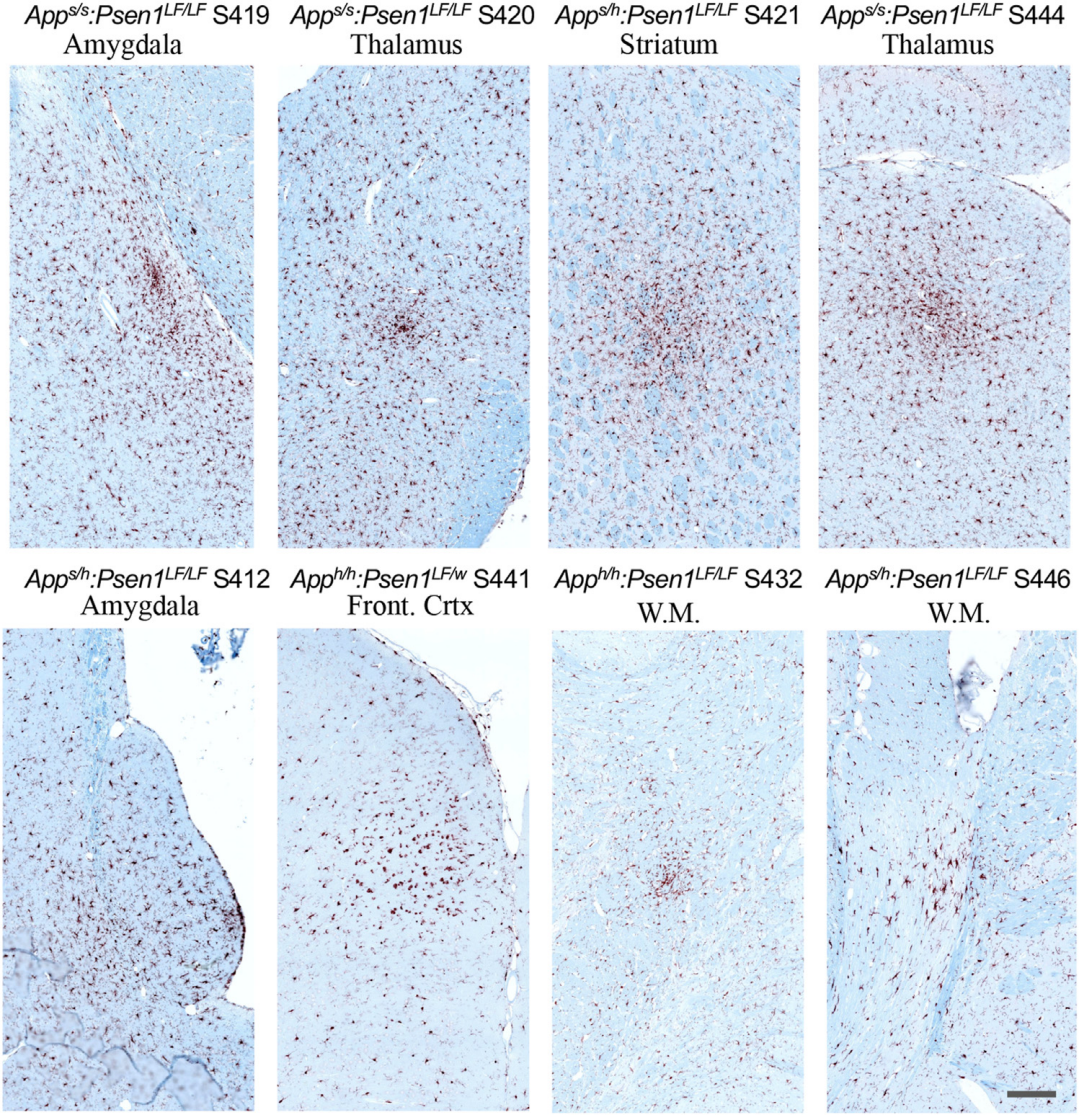
**Representative IBA1 microglial foci magnification in knock-in rats *App***^***s/s***^***:Psen1***^***LF/LF***^**S419, S420 and S444, *App***^***s/h***^***:Psen1***^***LF/LF***^**S412, S421 and 446, *App***^***h/h***^***:Psen1***^***LF/w***^**S441 and *App***^***h/h***^***:Psen1***^***LF/LF***^**S432.** Representative images of the IBA1 feature called “microglial foci” (*red-brown chromogen*) in knock-in rats. All pictures were taken at the same magnification; the scale bar is equivalent to 200 microns. APP, amyloid precursor protein; PSEN, Presenilin.

Overall, Iba1 staining did not reveal clear evidence of microglial inflammatory response related to the genotype, that is, a feature consistent in most of the samples of the group. A representative subject from each of the 14-month-old knock-in FAD and control groups is illustrated in Figure 2*A*. A magnified area of the CA1 illustrates similar activation levels within the hippocampus for the FAD knock-in rats compared to the control knock-in animals (Fig. 2*B*). Throughout the brain, the morphology of the microglia is generally ramified, without patches of bushy activated microglia or inflammatory foci, and both female and male cohorts have very similar Iba1 staining patterns. Blinded qualitative analysis indicated that, overall, there was no obvious difference in microglial activation within the FAD and control knock-in groups (Table 2). However, there were some brain areas with localized microglia activation in few subjects (summarized in Table 3). In the two subjects with CA1 focal degeneration (*App*^*s/h*^*:Psen1*^*LF/LF*^ S414 and *App*^*s/s*^*:Psen1*^*LF/LF*^ S426), microglia were highly activated in very close proximity to the degenerating CA1 (Fig. 3). Areas with microglial activation (defined as activated microglia spread apart in an area/part of a brain structure, Fig. 4) and microglial foci (defined as an area with activated microglia regrouped and seemingly attracted toward a focal point, Fig. 5) were observed in one *App*^*h/h*^*:Psen1*^*LF/w*^ (S441), one *App*^*h/h*^*:Psen1*^*LF/LF*^ (S432), four *App*^*s/h*^*:Psen1*^*LF/LF*^ (S421, S412, S446, S438) and four *App*^*s/s*^*:Psen1*^*LF/LF*^ (S419, S420, S444, S426) subjects, but not in *App*^*h/h*^*:Psen1*^*w/w*^ control samples. These areas were in various brain structures, including thalamus, white matter, amygdala, CA1/CA2, entorhinal cortex, frontal cortex, and striatum.

**Table 3:** Cases from which human brain slices were obtained

Sample ID#	Group	Braak stage	Age	Sex	Brain region
1010	MCI	4	91	M	Superior and middle temporal gyri
1130	MCI	5	99	F	Superior and middle temporal gyri
1204	AD	6	72	F	Temporal lobe
1222	AD	6	67	M	Temporal lobe
1290	MCI	4	93	F	Superior and middle temporal gyri
1300	MCI	5	91	M	Superior and middle temporal gyri
1319	MCI	5	94	M	Superior and middle temporal gyri
NA98-306-J	CTRL				Hippocampus/Entorhinal cortex
NA98-308-M	CTRL				Hippocampus/Entorhinal cortex

**Figure 6:**
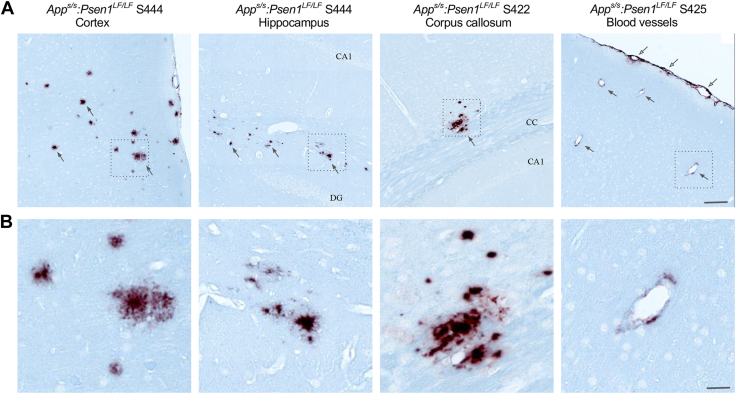
**Amyloid deposition examples in the cortex, hippocampus, corpus callosum, and blood vessels.***A*, representative staining of amyloid deposits, from *left* to *right*, in the cortex (*App*^*s/s*^*:Psen1*^*LF/LF*^ S444), the hippocampus (*App*^*s/s*^*:Psen1*^*LF/LF*^, S444), the corpus callosum (*App*^*s/s*^*:Psen1*^*LF/LF*^, S422), and blood vessels (*App*^*s/s*^*:Psen1*^*LF/LF*^, S425) (CA1: CA1 region of the hippocampus, DG: Dentate Gyrus, CC: Corpus Callosum). *Squares* indicate the area magnified in *row* (*B*), *arrows* indicate plaques or vessels deposit locations. *Open arrows* indicate leptomeningeal amyloid deposition. The scale bar is equivalent to 100 microns and 25 microns, respectively. APP, amyloid precursor protein; PSEN, Presenilin.

A 6E10 and 4G8 antibody mix was used to investigate the presence of amyloid plaques. A representative subject from each group is illustrated in Figure 2*A*. All 10 *App*^*s/s*^*:Psen1*^*LF/LF*^ animals presented amyloid plaques. The plaque morphology was usually round, small, dense, and they were present in small clusters. Plaques were slightly more numerous, larger, and more frequently located in the cortex (in 100% of *App*^*s/s*^*:Psen1*^*LF/LF*^) and randomly distributed across the cortical mantle; no particular subregion was more affected. A few small plaques were located in the hippocampus (in 80% of *App*^*s/s*^*:Psen1*^*LF/LF*^), more specifically between the dentate gyrus and CA1. Some plaques were also found in the corpus callosum (in 50% of *App*^*s/s*^*:Psen1*^*LF/LF*^) and finally, one plaque in the thalamus (in 20% of *App*^*s/s*^*:Psen1*^*LF/LF*^) (Fig. 6). In addition, strong to moderate amyloid deposition was also observed in the leptomeningeal blood vessel walls in 5 out of 10 *App*^*s/s*^*:Psen1*^*LF/LF*^ animals (see Fig. 6 and summary Table 2). Moderate to low deposition in the cortex vasculature of the brain was also observed in animals with leptomeningeal deposition (see Fig. 6). In the *App*^*s/h*^*:Psen1*^*LF/LF*^ group, 2 out of 12 animals had 2 to 3, very small amyloid plaques (S456, S415), and two animals had deposits in the leptomeningeal vessels (S414, S455). No 6E10/4G8 staining was observed in any animals from the other genotypes. No staining with AT8 was observed in any of the animals studied, consistent with the absence of silver staining–positive tangles. A representative animal from each group is illustrated in Figure 2*A*. The modified Bielshowsky’s silver stain was used to identify plaque structures in the tissue, along with aberrant neuronal inclusion such as tangles and apoptotic-driven cell death. A representative subject from each of the groups is illustrated in Figure 2*A*. A magnified area of the CA1 illustrates very similar structures within the hippocampus for the knock-in rats groups compared to the WT animals (Fig. 2*B*).

### Amyloid plaques of *App*^*s/s*^*:Psen1*^*LF/LF*^ and *App*^*s/h*^*:Psen1*^*LF/LF*^ knock-in rats are composed of Aβ40, Aβ42, and Aβ43

**Figure 7:**
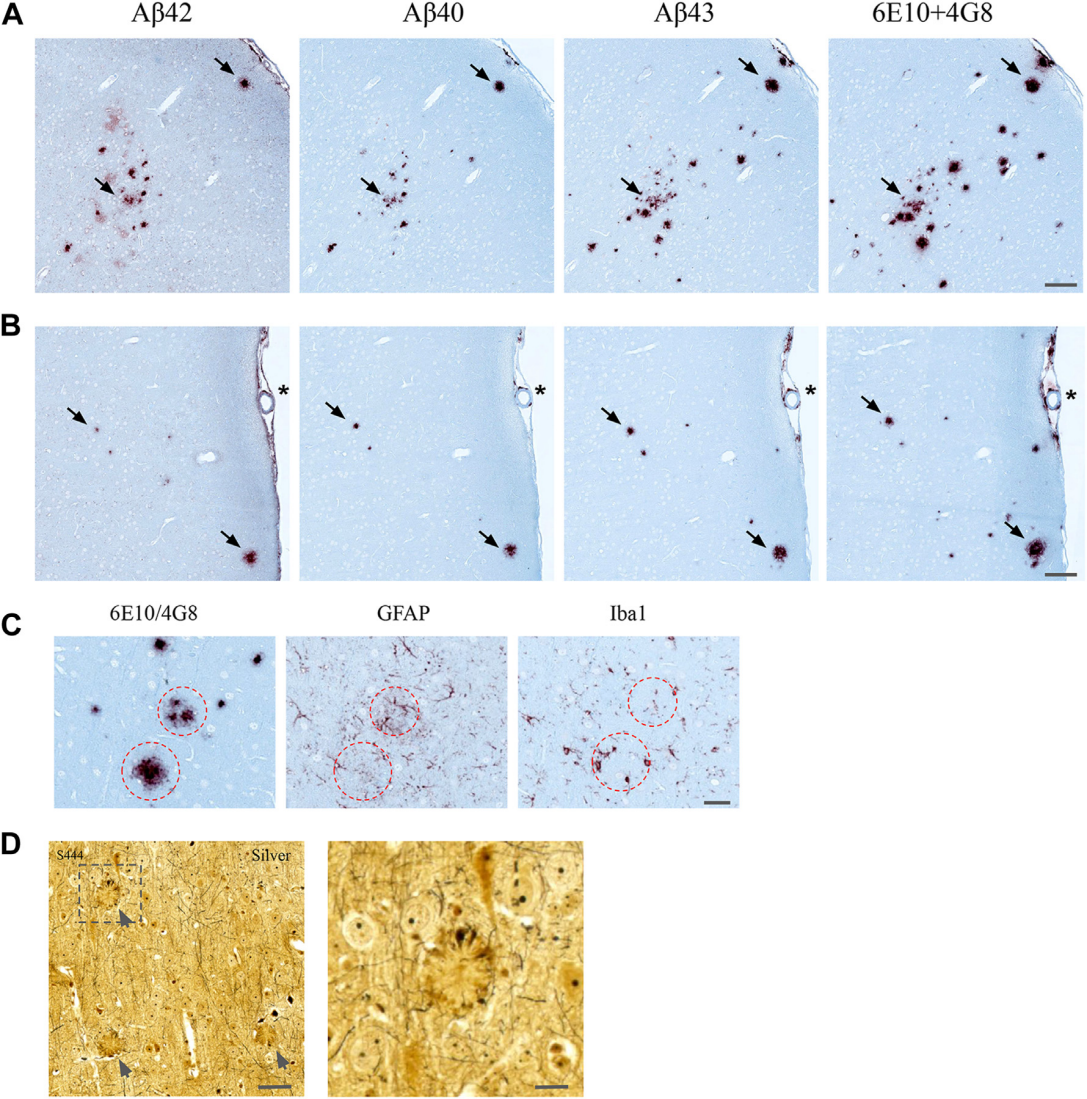
**Characterization of amyloid plaques.** Consecutive slides staining with Aβ42, Aβ40, Aβ43, and pan-Aβ (6E10/4G8 mix) in *App*^*s/s*^*:Psen1*^*LF/LF*^ S425 (*A*) and S444 (*B*) cortex. *Dark arrows* indicate amyloid plaque’s locations present on the four slides; note the presence of all the Aβ isoforms tested in the amyloid plaques. The *asterisk* in (*B*) indicates leptomeningeal amyloid deposition. Scale bar is equivalent to 100 μm. *C*, IBA1 and GFAP staining in the vicinity of amyloid plaques. Consecutive slides staining with 6E10/4G8, GFAP, and Iba1 in a region of the cortex with amyloid deposits (*App*^*s/s*^*:Psen1*^*LF/LF*^ S450). The scale bar is equivalent to 25 microns. *D*, representative images of amyloid plaques stained with silver staining in *App*^*s/s*^*:Psen1*^*LF/LF*^ S444. *Gray arrows* indicate the amyloid plaques, and the *square* is magnified on the right panel. The scale bar displayed is equivalent to 50 microns and 5 microns, respectively. Aβ, amyloid β; APP, amyloid precursor protein; PSEN, Presenilin.

Composition of the amyloid plaques was evaluated with antibodies directed against Aβ40, Aβ42, Aβ43, and the 6E10/4G8 mix (pan-Aβ). Antibodies directed against Aβ40, Aβ42, Aβ43 allow to identify the Aβ species forming amyloid plaques and, unlike 6E10 and 4G8, do not bind APP, APP-CTFs, and large sAPP molecules. Consecutive slices were stained in the following order Aβ42 > Aβ40 > Aβ43 > pan-Aβ (Fig. 7*A*). The samples stained were as follows: four *App*^*h/h*^*:Psen1*^*w/w*^ control animals, the two *App*^*s/h*^*:Psen1*^*LF/LF*^ animals with a few plaques (S456, S415), and all the *App*^*s/s*^*:Psen1*^*LF/LF*^ animals. Plaques appeared positive for all the Aβ isoforms studied in all the animals studied, that is, when a plaque was spanning across the four slides, it was positive for Aβ40, Aβ42, and Aβ43. Location of the plaques did not appear to affect its composition, that is, all the Aβ isoforms were present in plaques located in the cortex, hippocampus, or corpus callosum. It appeared that Aβ40 and Aβ42 were mainly present in the center of the plaques, whereas Aβ43 was present in the center and border of the plaques (Fig. 7*B*). No Aβ40, Aβ42, Aβ43, or pan-Aβ was observed in the *App*^*h/h*^*:Psen1*^*w/w*^ control animals. The very small plaques from the two *App*^*s/h*^*:Psen1*^*LF/LF*^ S456 and S415 were also positive for all the Aβ isoforms tested.

To assess the presence of activated astrocytes and microglia in the vicinity of amyloid plaques, we analyzed consecutive 6E10/4G8, GFAP, and Iba1-stained slides. A few mildly activated astrocytes and microglia were found surrounding the largest plaques; their low number and moderately activated morphology suggested relatively young plaque formation (see Fig. 7*C*). Therefore, plaque-induced microglial activation was present but limited.

Silver-stained plaque formations were not as abundant as the amyloid staining and were not as frequently observed in *App*^*s/s*^*:Psen1*^*LF/LF*^ tissues. When present, the silver staining of amyloid plaques appeared light and not as dark as mature plaques are usually observed in the literature. Only the largest, rare, round plaques in the cortex could be observed, clusters of small plaques were not visible with silver staining. Plaque locations were more apparent due to darker circular staining circumscribing the periphery of the plaque rather than the plaque themselves (see Fig. 7*D*). Together, these observations suggest relatively young plaques, which is consistent with the small plaque size and the mild microglia and GFAP activation surrounding them. We did not observe neurons bearing tau tangles in the surroundings of the plaques nor anywhere else in the brain.

### Knock-in rats that develop amyloid pathology have highest CNS Aβ43 levels

The evidence that 14-months-old *App*^*s/s*^*:Psen1*^*LF/LF*^ have a low number of young plaques and that only ∼16% of *App*^*s/h*^*:Psen1*^*LF/LF*^ animals showed 2 to 3 small plaques indicates recent amyloid deposition in these rats. Thus, to measure CNS Aβ species composition that is needed to initiate amyloid deposition, we performed Aβ ELISA measurements in 7-month-old rats. To produce rats carrying all possible genetic permutations of these two mutants, we crossed male and female *App*^*s/h*^*:Psen1*^*LF/w*^ rats. Animals of all nine possible genetic combinations were obtained: *App*^*h/h*^*:Psen1*^*w/w*^, *App*^*s/h*^*:Psen1*^*w/w*^, *App*^*s/s*^*:Psen1*^*w/w*^*, App*^*h/h*^*:Psen1*^*LF/w*^, *App*^*s/h*^*:Psen1*^*LF/w*^, *App*^*s/s*^*:Psen1*^*LF/w*^, *App*^*h/h*^*:Psen1*^*LF/LF*^, *App*^*s/h*^*:Psen1*^*LF/LF*^, and *App*^*s/s*^*:Psen1*^*LF/LF*^.

**Figure 8:**
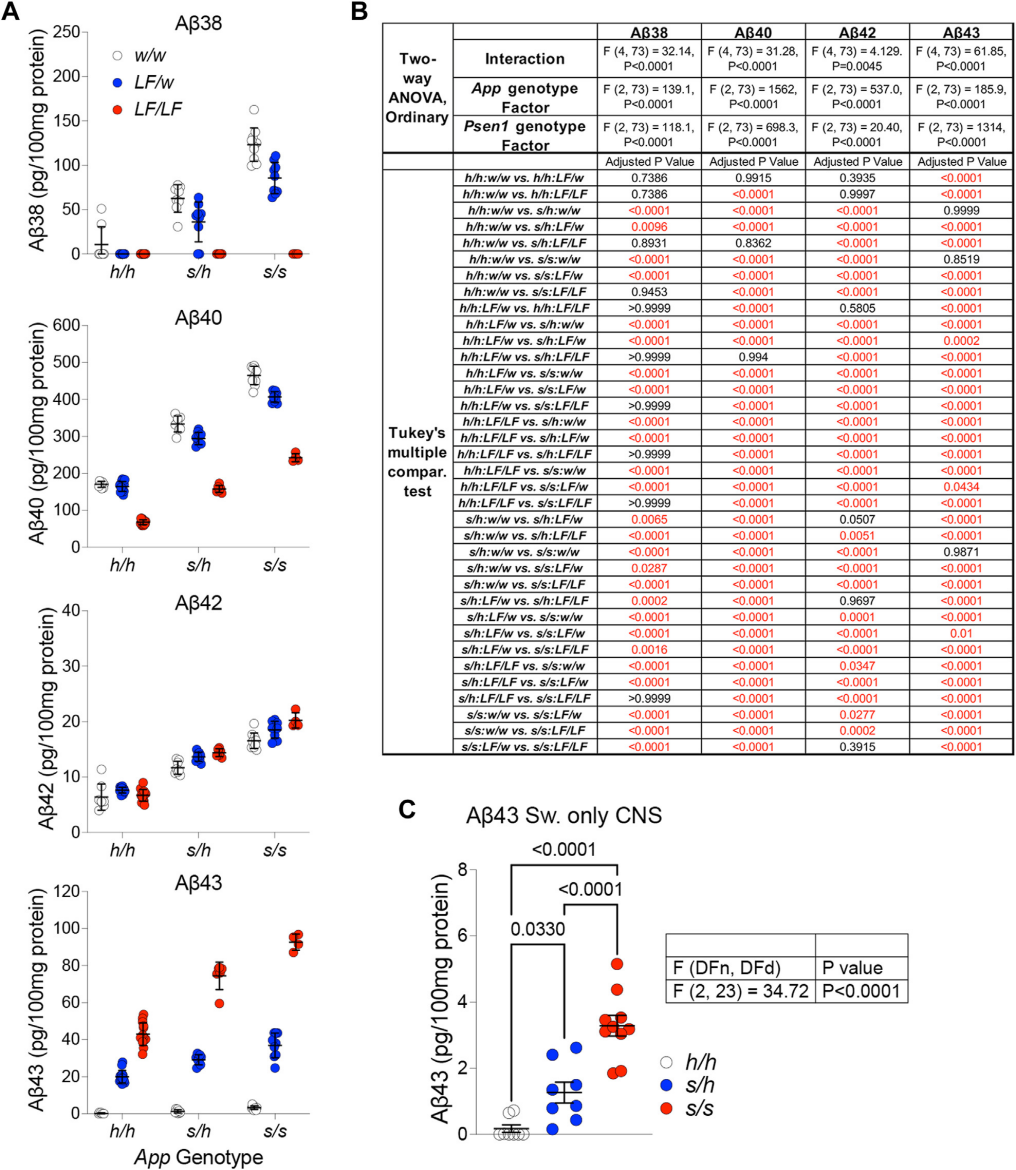
**Levels of Aβ38, Aβ40, Aβ42, and Aβ43 in the CNS of 7-month-old knock-in rats carrying *App***^***s***^**and *Psen1***^***LF***^**FAD mutations.***A*, Aβ38, Aβ40, Aβ42, and Aβ43 were measured in rats of the following genotypes (number of female and male rats used are indicated in parentheses): *App*^*h/h*^*:Psen1*^*w/w*^ (4 females and 4 males), *App*^*s/h*^*:Psen1*^*w/w*^ (4 females and 4 males), *App*^*s/s*^*:Psen1*^*w/w*^ (6 females and 4 males), *App*^*h/h*^*:Psen1*^*LF/w*^ (9 females and 5 males), *App*^*s/h*^*:Psen1*^*LF/w*^ (4 females and 5 males), *App*^*s/s*^*:Psen1*^*LF/w*^ (4 females and 5 males), *App*^*h/h*^*:Psen1*^*LF/LF*^ (5 females and 7 males), *App*^*s/h*^*:Psen1*^*LF/LF*^ (2 females and 2 males), and *App*^*s/s*^*:Psen1*^*LF/LF*^ (1 female and 3 males) knock-in rats. Data are represented as mean ± S.D. and were analyzed by ordinary two-way ANOVA followed by ad hoc Tukey’s multiple comparison test when ANOVA shows significant differences. Detailed statistical analysis is reported in (*B*), with significant difference reported in *red*. *C*, ordinary one-way ANOVA (F (2, 23) = 34.72, *p* < 0.0001) and Tukey’s multiple comparison test comparing Aβ43 levels of *App*^*h/h*^*:Psen1*^*w/w*^, *App*^*s/h*^*:Psen1*^*w/w*^, and *App*^*s/s*^*:Psen1*^*w/w*^ knock-in rats. The *p* values of the three comparisons are reported in the panel. Aβ, amyloid β; APP, amyloid precursor protein; FAD, Familial forms of Alzheimer’s disease; PSEN, Presenilin.

First, we determined if and how the interaction of these two pathogenic mutations alter Aβ species profiles. Brains were harvested from ∼7-months-old rats, and levels of Aβ38, Aβ40, Aβ42, and Aβ43 were measured by ELISA. These four Aβ species are the products of the third and fourth catalytic steps of product line 1 (Aβ43 and Aβ40) and product line 2 (Aβ42 and Aβ38). All data relating to these experiments are shown in Figure 8*A*. The detailed statistical analyses are shown in Figure 8*B*. Aβ38 was barely detectable in control animals, undetectable in *App*^*h/h*^*:Psen1*^*LF/w*^ and *App*^*h/h*^*:Psen1*^*LF/LF*^ rats, and augmented in Swedish rats in a gene-dosage–dependent manner. The Swedish mutation-dependent increase in Aβ38 was significantly reduced by one *Psen1*^*LF*^ allele and was occluded by two *Psen1*^*LF*^ alleles. A similar pattern was observed for Aβ40, which was increased and decreased in a gene-dosage–dependent manner by the *App*^*s*^ and *Psen1*^*LF*^ mutations, respectively. The *Psen1*^*LF*^ allele significantly reduced, in a gene-dosage–dependent manner; the increase in Aβ40 caused by the Swedish mutation. Aβ42 levels were increased by the Swedish mutation in a gene-dosage–dependent manner but were not significantly altered by the *Psen1*^*LF*^ mutation. Yet, the *Psen1*^*LF*^ mutation further boosted the increase in Aβ42 levels caused by *App*^*s*^. A gene-dosage–dependent increase in Aβ43 levels was observed in rats carrying the *Psen1*^*LF*^ mutation. Aβ43 levels were higher than control in both heterozygous and homozygous *App*^*s*^ rat, but the increase was not statistically significant in this two-way ANOVA analysis. One-way ANOVA analysis of *App*^*h/h*^*:Psen1*^*w/w*^, *App*^*s/h*^*:Psen1*^*w/w*^, and *App*^*s/s*^*:Psen1*^*w/w*^ samples showed a gene-dosage–dependent increase in Aβ43 in *App*^*s*^ rats, when in combination with one or both *Psen1*^*LF*^ alleles; however, given the large effect size of the *Psen1*^*LF*^ allele on Aβ43 production, when you analyze the *Psen1*^*w*^-only animals separately, a statistically significant increase in Aβ43 caused by the *App*^*s*^ allele is evident as well (Fig. 8*C*). In line with these observations, we found a synergistic effect of the two pathogenic mutations on Aβ43 levels, with exceptionally high levels of Aβ43 in *App*^*s/h*^*:Psen1*^*LF/LF*^ and, yet more, in *App*^*s/s*^*:Psen1*^*LF/LF*^ rats.

**Figure 9:**
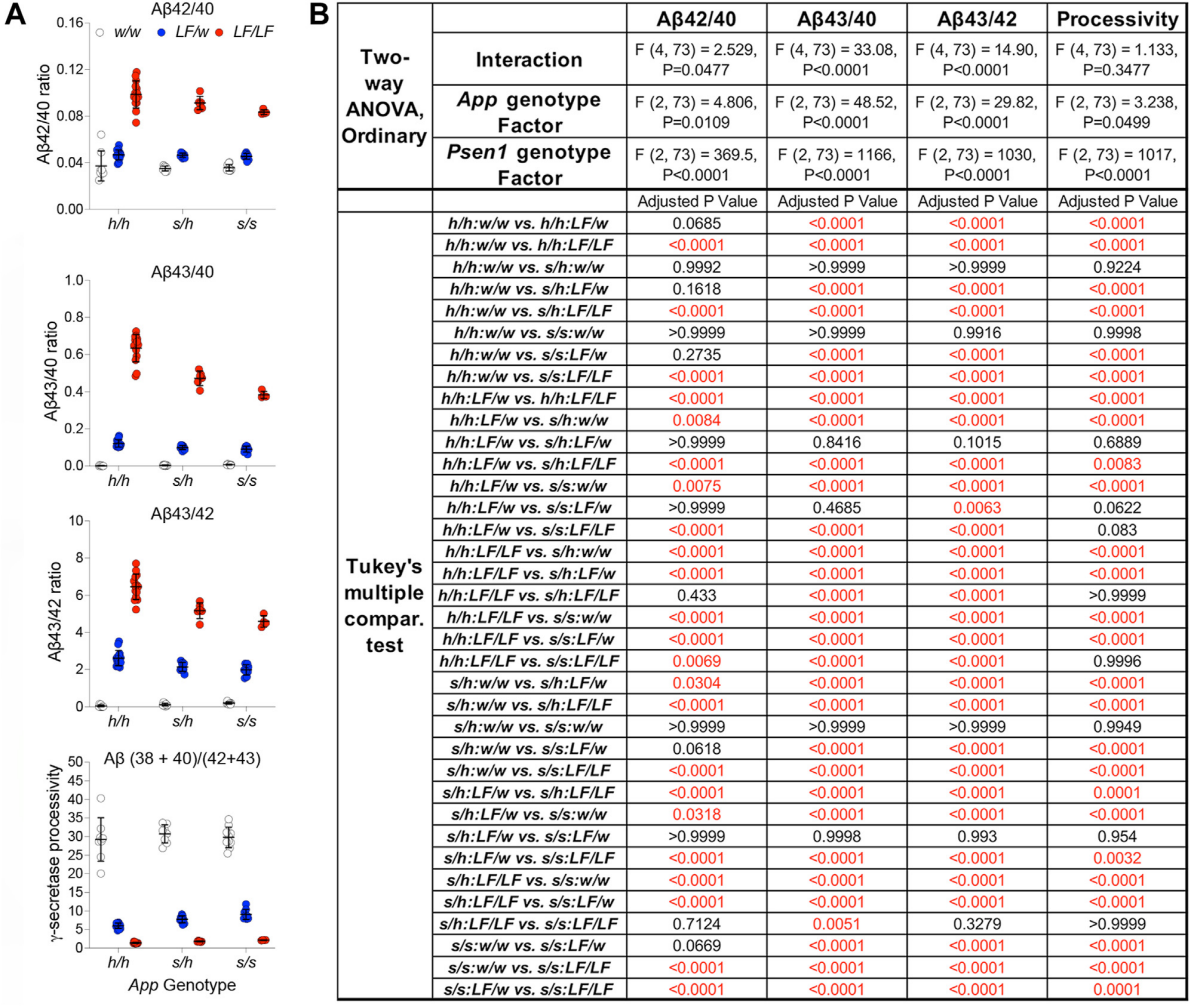
**Aβ42/Aβ40 ratios, Aβ43/Aβ40 ratios, Aβ43/Aβ42 ratios, and γ-secretase processivity -(Aβ (38 + 40)/(42 + 43) ratios-in the CNS of 7-month-old knock-in rats carrying *App***^***s***^**and *Psen1***^***LF***^**FAD mutations.***A*, Aβ42/Aβ40 ratios, Aβ43/Aβ40 ratios, Aβ43/Aβ42 ratios, and γ-secretase processivity -(Aβ (38 + 40)/(42 + 43) ratios- were measured using the data shown in Figure 2*A*. Data are represented as mean ± S.D. and were analyzed by ordinary two-way ANOVA followed by ad hoc Tukey’s multiple comparison test when ANOVA shows significant differences. Detailed statistical analysis is reported in (*B*), with significant difference reported in *red*. Aβ, amyloid β; APP, amyloid precursor protein; FAD, Familial forms of Alzheimer’s disease; PSEN, Presenilin.

As discussed above, increase in the Aβ42/Aβ40 ratio is considered a predictor of amyloid pathology and AD. This ratio was increased by the *Psen1*^*LF*^ mutation in a gene-dosage–dependent manner but was unaffected by the Swedish mutation. Moreover, the Swedish mutation reduced the increase of these ratios caused by the *Psen1*^*LF*^ mutation in a gene-dosage–dependent manner. We extended this analysis to the Aβ43/Aβ40 and Aβ43/Aβ42 ratios and observed identical patterns. All data relative to these experiments are shown in Figure 9*A*, with the detailed statistical analyses shown in Figure 8*B*.

To quantify γ-secretase processivity, Chávez-Gutiérrez’s lab has introduced a ratio between the sum of the products of the fourth catalytic turnover divided by the sum of the products of the third catalytic step, which are the substrates of the fourth catalytic turnover (9). Ultimately, this ratio provides an overall measure of γ-secretase processivity along both product lines. Applying this method to an *in vitro* analysis of Aβ profiles of 25 FAD-linked PSEN1 mutants, the authors found a linear correlation between mutation-driven alterations in Aβ profiles and age at onset of AD in humans (9). Hence, to assess γ-secretase processivity in our nine rat lines, we calculated the Aβ (38 + 40)/(42 + 43) ratios. We observed a gene-dosage–dependent reduction of γ-secretase processivity caused by the *Psen1*^*LF*^ mutation; in contrast, the Swedish mutation did not significantly change γ-secretase processivity, regardless of the *Psen1* genotype (Fig. 9, *A* and *B*).

Overall, the data indicate that co-expression of the *Psen1*^*LF*^ and the Swedish mutations cause both synergistic and opposite effects on Aβ brain composition in a gene-dosage–dependent manner: Aβ43 production is synergistically increased by the two FAD mutations, while the *Psen1*^*LF*^ mutation obliterates Aβ38 production, which is increased by the Swedish mutation.

### Aβ43 is found in the core of amyloid plaques of LOAD and MCI cases

**Figure 10:**
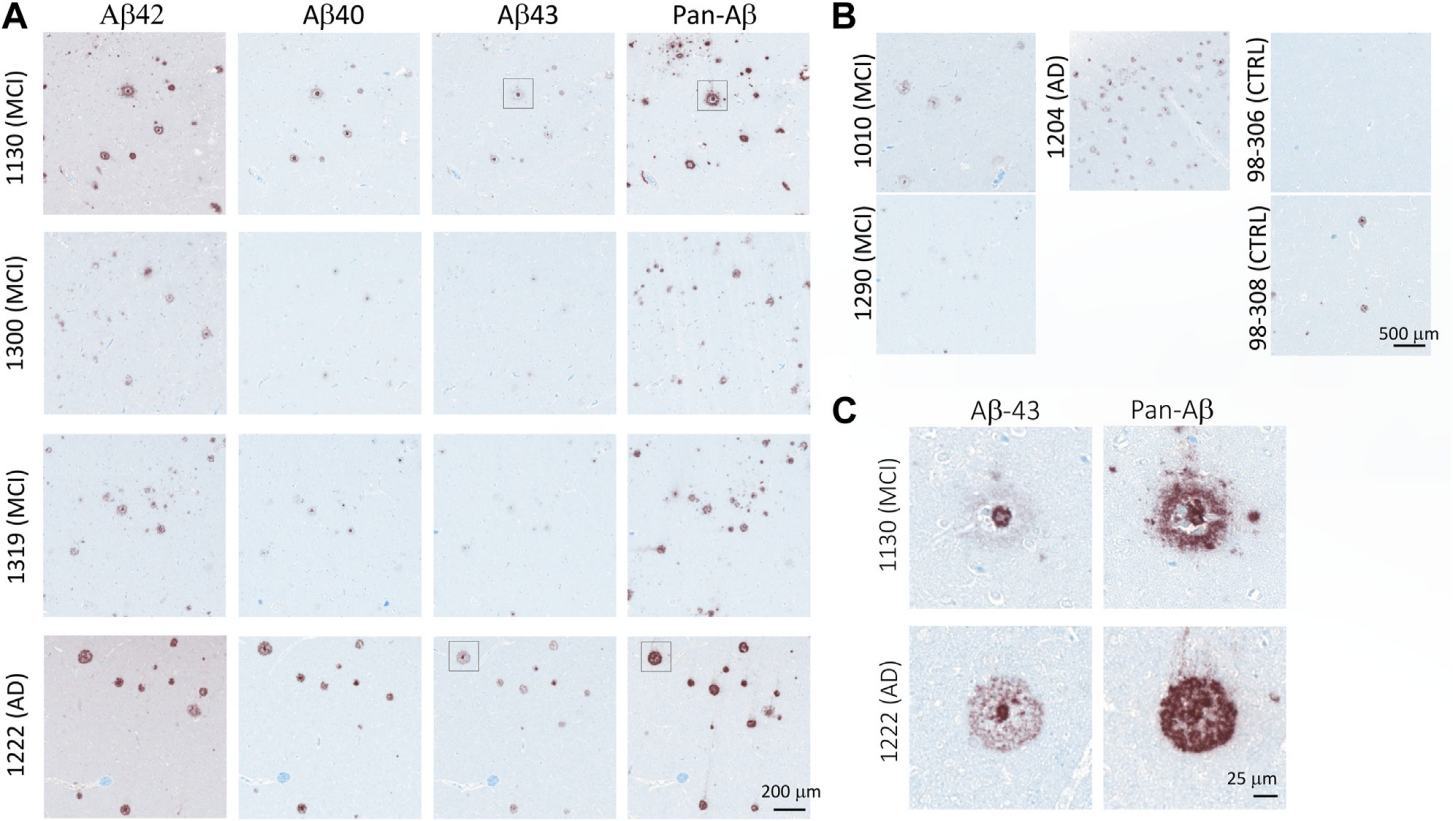
**Amyloid deposition examples in the cortex of MCI and LOAD subjects**. *A*, representative staining of amyloid deposits, detected from *left* to *right*, with anti-Aβ42, anti-Aβ40, anti-Aβ43, and anti-Pan-Aβ antibodies. Stainings were performed on consecutive slices collected in that same order. Subjects 1130, 1300, 1319 are classified as MCI and 1222 as LOAD. The scale bar displayed is equivalent to 200 microns. *B*, representative staining of amyloid deposits, detected with the anti-Aβ43 antibody on additional MCI (1010,1290), LOAD (1204), and control subjects (98–306, 98–308). The scale bar displayed is equivalent to 500 microns. *C*, high magnification (the scale bar displayed is equivalent to 25 microns) of amyloid plaques stained with anti-Aβ43 and anti-Pan-Aβ antibodies in both MCI (1130) and LOAD (1222) showing Aβ43 staining in the plaque core. These images represent magnification of the plaques surrounded by a *square* in 10A. Aβ, amyloid β.

To test whether these rat knock-in models can inform about disease mechanisms driving amyloid pathology in sporadic LOAD cases, we analyzed amyloid plaques composition in MCI and LOAD cases (Table 3). Consecutive slices from three MCI and one LOAD cases were stained, with the same antibodies used for rat IHC, in the following order Aβ42 > Aβ40 > Aβ43 > pan-Aβ (Fig. 10*A*). Like for *App*^*s/h*^*:Psen1*^*LF/LF*^ and *App*^*s/s*^*:Psen1*^*LF/LF*^ animals, plaques were positive for all the Aβ isoforms studied. We also stained for the Aβ43 single brain slice available from five more cases (2 MCI, 1 LOAD, and 2 controls) and found Aβ43+ plaques in all three disease cases Figure 10*B*). Interestingly, one of the two controls (#98-308) also showed Aβ43+ plaques (Fig. 3*B*, right-bottom panel). Notably, Aβ43 appears to be concentrated in the plaques core (Fig. 10*C*).

## Discussion

Understanding the changes in the molecular composition of Aβ profiles that are needed to initiate amyloid deposition is a central question in the context of the amyloid hypothesis of AD pathogenesis. To provide insights into the composition of pathogenic Aβ cocktails, we have studied knock-in rats expressing eight permutations of the FAD *APP* Swedish and *PSEN1 L435F* mutations. The APP mutation’s primary effect is to increase the levels of the γ-secretase substrate and Aβ precursor APP-βCTF. This leads to a gene-dosage–dependent increase in all Aβ species analyzed (*i.e.*, Aβ38, Aβ40, Aβ42, and Aβ43). In contrast, the *PSEN1 L435F* decreases the activity and processivity of γ-secretase. As a result, this mutation causes a gene-dosage–dependent shift from shorter to longer Aβ species leading to a decrease in total Aβ amounts. This shift is dramatically obvious for the Aβ product line 1, as shown by the large increase in Aβ43, generated by the third catalytic step, concurrent with a large decrease in Aβ40, which is derived from Aβ43 in the fourth catalytic step. Coexistence of the two mutations in the same subject causes both synergistic and opposite effects on Aβ brain composition. For example, the two pathogenic mutations synergistically increase Aβ43 levels in a gene-dosage–dependent manner. In contrast, the *Psen1*^*LF*^ allele significantly reduces, also in a gene-dosage–dependent manner; the increase in Aβ40 and Aβ38 caused by the Swedish mutation. These changes can be rationally explained by the expression of γ-secretase with reduced activity/processivity concurrent with increased availability of APP-βCTF.

IHC analysis shows that, at 14 months of age, 100% (10 out of 10) of *App*^*s/s*^*:Psen1*^*LF/LF*^ rats develop amyloid pathology. Plaques are seen in several brain regions, including the cortical mantle, hippocampus, corpus callosum, and thalamus. In 50% of the animals, amyloid deposition is also observed in the leptomeningeal blood vessel walls. Plaques are composed by Aβ40, Aβ42, and Aβ43, with Aβ40 and Aβ42 mainly present in the center of the plaques and Aβ43 present in the center and border of the plaques. Plaques are mostly round, small, and dense, with few larger plaques that are mainly located in the cortex. In silver staining, amyloid plaques appear light, which contrasts the dark aspect of mature plaques. A few mildly activated microglia surround the largest plaques. Altogether, these observations suggest relatively young plaque formation. Two out of twelve *App*^*s/h*^*:Psen1*^*LF/LF*^ animals (16.6%) had 2 to 3, very small amyloid plaques, and two animals had deposits in the leptomeningeal vessels. No amyloid plaques are detected in the other genotypes analyzed at either 14 (*App*^*h/h*^*:Psen1*^*LF/w*^, *App*^*h/h*^*:Psen1*^*LF/LF*^) or 18 (*App*^*s/h*^*:Psen1*^*w/w*^, *App*^*s/s*^*:Psen1*^*w/w*^*, App*^*s/h*^*:Psen1*^*LF/w*^ and *App*^*s/s*^*:Psen1*^*LF/w*^) months of age. In summary, development of amyloid pathology requires co-expression of two *Psen1*^*LF*^ mutant alleles with at least one *App*^*s*^ mutant allele: doubling the *App*^*s*^ load significantly accelerated amyloid deposition.

The evidence that 14-months-old *App*^*s/s*^*:Psen1*^*LF/LF*^ have a low number of relatively young plaques and that only ∼16% of *App*^*s/h*^*:Psen1*^*LF/LF*^ animals showed 2 to 3 small plaques indicates that the ELISA measurements performed in 7-month-old rats reflect prepathological CNS Aβ species composition needed to initiate amyloid deposition. Comparing the genetic makeup leading to amyloid pathology with the quantification of Aβ species leads to the following conclusions. 1) Aβ42/Aβ40, Aβ43/Aβ40, and Aβ43/Aβ42 ratios values follow this hierarchy: *App*^*h/h*^*:Psen1*^*LF/LF*^ > *App*^*s/h*^*:Psen1*^*LF/LF*^ > *App*^*s/s*^*:Psen1*^*LF/LF*^. Yet, *App*^*h/h*^*:Psen1*^*LF/LF*^ show no plaques, *App*^*s/h*^*:Psen1*^*LF/LF*^ show few plaques in 16% of the subjects, while *App*^*s/s*^*:Psen1*^*LF/LF*^ show more plaques in 100% of the subjects analyzed. Thus, early amyloid plaques formation is not associated with highest long-Aβ/short-Aβ ratios. In fact, 2) Aβ42 levels are significantly higher in *App*^*s/s*^*:Psen1*^*LF/w*^ as compared to *App*^*s/h*^*:Psen1*^*LF/LF*^ animals (*p* < 0.0001). Yet, *App*^*s/h*^*:Psen1*^*LF/LF*^ rats start showing signs of plaques deposition at 14 months of age, while *App*^*s/s*^*:Psen1*^*LF/w*^ animals do not show any amyloid plaques even at 18 months of age. 3) Amyloid plaques are detected in the genotypes with highest levels of Aβ43 (*App*^*s/s*^*:Psen1*^*LF/LF*^ and *App*^*s/h*^*:Psen1*^*LF/LF*^ animals), in a manner proportional to Aβ43 levels (Aβ43 levels: *App*^*s/s*^*:Psen1*^*LF/LF*^ *> App*^*s/h*^*:Psen1*^*LF/LF*^, *p* < 0.0001). Although a complete analysis of all Aβ species generated along products line 1 (Aβ49 → Aβ46 → Aβ43 → Aβ40 → Aβ37, which is a minor product of a fifth catalytic step) and 2 (Aβ48 → Aβ45 → Aβ42 → Aβ38) would make this analysis exhaustive, the data reported here indicate that the CNS levels of Aβ43, rather than Aβ species’ ratios and absolute levels of Aβ42, determine the speed of pathological amyloid deposition in the knock-in rats studied.

Finally, the evidence that Aβ43 is found in in the amyloid plaque’s core of all LOAD and MCI cases we studied is consistent with the idea that Aβ43-containing aggregates act as seeds that catalyze amyloid plaque formation.

Several studies have shown that FAD mutations cause a significant increase in Aβ43 levels. These studies include analysis of PSEN1-R278I in *Presenilins* KO mouse embryonal fibroblast (33), PSEN1-R278I, PSEN1-C410Y, and PSEN1-L435H in HEK cells and mouse embryonal fibroblasts (24, 34, 35), PSEN1-V261F, PSEN1-R278I, PSEN1-L435F, PSEN1-L166P, PSEN1-Y256S, G382A in HEK293/swe cells (36), PSEN1-R278I (33), PSEN1-I213T (37), and PSEN1-P117L (38) in knock-in mice, PSEN1-L435F patient-derived iPSC neurons (39). Moreover, Aβ43 has also been found in amyloid plaque lesions of seven cases with the APP Swedish mutation and three cases with the PSEN1-I143T FAD mutation (40, 41, 42), as well as two cases with the PSEN1-L435F mutation (24). Most importantly, the presence of Aβ43 in amyloid plaques of LOAD cases was previously described also in other studies (24, 40, 42), and CSF level of Aβ43 is a significant predictor of MCI and cerebral amyloid deposits (43). Moreover, a 2-years follow up study showed that CSF levels of Aβ43, not Aβ42, decreased in patients who progressed from MCI patients to sporadic AD (44), suggesting a faster rate of Aβ43 deposition as compared to Aβ42 deposition. Interestingly, the mean age of onset in patients carrying the Swedish mutation is 55 years with a range of 45 to 61 years, and the mean duration of the illness is 7 years (45). In contrast, the average age of onset in patients carrying the *PSEN1-L435F* mutation is 47 years, and the average age at death is 56 years (29). The higher aggressiveness of the *PSEN1-L435F* mutation associates with the significantly higher levels of Aβ43 production detected in *Psen1*^*LF*^ rats compared to *App*^*s*^ rats. Altogether, the above data support the hypothesis that Aβ43 could play a major role in determining the onset of pathological amyloid deposition in both familial and sporadic AD.

In conclusion, this study corroborates the critical pathological importance of alterations in the Aβ peptides composition, helps clarifying the molecular determinants initiating amyloid pathology, and supports therapeutic interventions targeting Aβ43 to prevent, delay, or revert AD.

## Experimental procedures

### Experimental animals

All experiments were done according to policies on the care and use of laboratory animals of the Ethical Guidelines for Treatment of Laboratory Animals of the NIH. Relevant protocols were approved by the Rutgers Institutional Animal Care and Use Committee (Protocol #201702513). All efforts were made to minimize animal suffering and reduce the number of rats used.

### Rat genotyping

The genotype of rats was verified by DNA sequencing of genomic DNA PCR products as previously reported (5, 26). To prepare genomic DNA, tail tissue was digested in 300 μl lysis buffer (100 mM Tris, 5 mM EDTA, 0.2% SDS, 200 mM NaCl, pH 8.0) plus 3 μl of 20 μg/ml protease K at 55 °C overnight. One hundred microliters of a 7.5 M ammonium acetate solution was added to each sample to precipitate protein; samples were mixed by vortexing for 30 s and centrifuged at 15000*g* for 5 min. Supernatant was mixed with 300 μl isopropanol and centrifuged at 15,000*g* for 5 min to precipitate genomic DNA. The DNA pellets were desalted with 70% ETOH and were dissolved in water for PCR and sequencing.

### Immunohistochemistry

Rat brain tissue was fixed and stored in 70% ethanol after transcardiac perfusion with PBS and 4% paraformaldehyde fixative. All tissues were dehydrated through graded ethanol and xylene, infiltrated with paraffin wax, and embedded in paraffin blocks. Sections were cut on a rotary microtome at the thickness of 5 μm, floated on a water bath, and mounted on glass slides. Slides were manually deparaffinized and rehydrated before the automated IHC. Slides initially underwent antigen retrieval, by one of the following methods, heat-induced epitope-retrieval or formic acid (FA) treatment. Heat-induced epitope-retrieval was performed by incubation in a citrate buffer (pH 6.0) (Abcam, ab93678) and heating to 100 °C for a period of 60 min. FA treatment was a 10-min incubation in 80% FA (Sigma, F0507), followed by washing in tris-buffered saline-Tween 20. All IHC studies were performed at room temperature on a Lab Vision Autostainer 360 (Thermo Fisher Scientific). Briefly, slides were incubated sequentially with hydrogen peroxide for 5 min, to quench endogenous peroxidase, followed by 5 min in a protein block (Abcam, ab156024) and then incubated with primary antibodies (see Table 4) in antibody diluent (Abcam, ab64211). Antibody binding was amplified using the appropriate secondary reagents (Jackson) (20 min), followed by a horseradish peroxidase conjugate (Jackson) (20 min) and visualized using the aminoethyl carbazole chromogen (Abcam, ab64252) (20 min). All IHC sections were counterstained with Acid Blue 129 (Sigma, 306496) and mounted with an aqueous mounting medium.

**Table 4:** Primary and amplification antibodies used for IHC

Target	Antibody	Antigen retrieval	Dilution	Secondary & amplification
Neurons	NeuN, Mouse monoclonal A60, Millipore	Citrate HIER	1:3000	RbαM & GtαRb-HRP
Amyloid β	1-16 and 17-24 Amyloid β Mouse monoclonal 6E10 and 4G8, Biolegend	80% Formic acid	1:1000 1:1000	RbαM & GtαRb-HRP
Microglia	Iba1, Rabbit polyclonal, Wako	Citrate HIER	1:200	GtαRb-HRP
Astrocytes	GFAP, Rabbit polyclonal, Thermo Fisher Scientific	Citrate HIER	1:200	DkαRb-bio & SA-HRP
Phospho-tau	Phospho-tau, AT8, Mouse monoclonal, Thermo Fisher Scientific	Citrate HIER	1:1000	HαM-bio & SA-HRP
Abeta40	Amyloid Beta 40, Mouse monoclonal G2-10, Millipore	80% Formic acid	1:500	HαM-bio & SA-HRP
Abeta42	Amyloid Beta 42, Mouse monoclonal G2-11, Millipore	80% Formic acid	1:500	HαM-bio & SA-HRP
Abeta43	Amyloid Beta 43, Rabbit polyclonal, IBL-America	80% Formic acid	1:100	DkαRb-bio & SA-HRP

Abbreviations: ∝, anti; bio, biotin; Ck, Chicken; Dk, Donkey; Gt, Goat; H, Horse; HIER, Heat induced antigen retrieval; HRP, Horseradish Peroxidase; M, Mouse; Rb, Rabbit; SA, Streptavidin.

Modified Bielshowsky’s Silver Staining: The slides were manually deparaffinized and rehydrated prior to histological staining. Rehydrated tissue was immersed in preheated silver nitrate solution (40 °C) for 15 min, followed by a deionized water rinse and an incubation in ammoniacal silver solution at 40 °C for 10 min (American Master Tech). Silver deposition was performed in the developer solution for a period of 15 min, once a golden-brown tissue stain was achieved; the development was stopped by sequential incubations in ammonium water and then 5% sodium thiosulfate (American Master Tech). The stained tissue sections were dehydrated in xylene and mounted in Permount (VWR) and coverslipped.

Qualitative image analysis of IHC Sections: The IHC and histology slides were digitized using an Axio Scan.Z1 digital whole slide scanner (Carl Zeiss). The images underwent quality control review and final images transferred to the Biospective server for qualitative image analysis. All qualitative assessments were performed blinded to the tissue’s genotype.

Human AD and MCI brain tissues were obtained from Dr Peter Nelson, ADC University of Kentucky (project financed by 10.13039/100000002NIH Grant P30 AG072946). The two control brain tissues were obtained from Archival material from Albert Einstein College of Medicine, courtesy of Dr Sunhee Lee. After a manual deparaffinization and rehydration, the same FA retrieval protocol as for the rat tissue was performed (80% FA for 10 min). The staining was performed on a Leica BOND-RX Staining System. The same primary antibodies and antibody concentration was used as for the rat tissue. The anti-Aß42, anti-Aß40, and 6E10+4G8 antibodies were incubated for 30 min, amplified using the polymer from the BOND kit (BOND Polymer Refine HRP PLEX Detection Kit, Cat no. DS9914) for 15 min and anti-Aß43 was incubated for 60 min and amplified 30 min with the BOND polymer. Staining was visualized using AEC Single Solution and mounted with an aqueous mounting medium.

### Rat brain proteins preparation and ELISAs

These procedures were performed as previously described (26, 46, 47, 48, 49, 50). For brain protein preparations, rats were anesthetized with isoflurane and perfused *via* intracardiac catheterization with ice-cold PBS. Brains were extracted and homogenized with a glass-teflon homogenizer in 250 mM sucrose, 20 mM Tris-base pH 7.4, 1 mM EDTA, 1 mM EGTA plus protease and phosphatase inhibitors (Thermo Fisher Scientific). All steps were carried out on ice. Homogenates were solubilized with 1% NP-40 for 30 min rotating and spun at 20,000*g* for 10 min. Supernatants were collected, and protein content was quantified by Bradford.

Aβ38, Aβ40, and Aβ42 were measured with V-PLEX Plus Aβ Peptide Panel 1 6E10 (K15200G). Measurements were performed according to the manufacturer’s recommendations. Plates were read on a MESO QuickPlex SQ 120. For analysis of Aβ43, IBL Human Amyloid β (1, 2, 3, 4, 5, 6, 7, 8, 9, 10, 11, 12, 13, 14, 15, 16, 17, 18, 19, 20, 21, 22, 23, 24, 25, 26, 27, 28, 29, 30, 31, 32, 33, 34, 35, 36, 37, 38, 39, 40, 41, 42) (FL) Assay Kit (27710) was used according to the manufacturer’s recommendations.

### Statistical analysis

Data were analyzed using GraphPad Prism software (www.graphpad.com) and expressed as mean ± S.D. Statistical tests used to evaluate significance and statistical data are shown in figures. Significant differences were accepted at *p* < 0.05.

## Data availability

The datasets used and/or analyzed during the current study are available from the corresponding author on reasonable request.

## Conflicts of interest

The authors declare that they have no conflicts of interest with the contents of this article.
